# Over-the-counter Medication Use among Patients Presenting with Fever in the Emergency Department in a Tertiary Care Hospital

**DOI:** 10.31729/jnma.8401

**Published:** 2024-01-31

**Authors:** Mrikchhya Ghimire, Ujjwol Prasad Risal, Rabin Bhandari

**Affiliations:** 1 Department of General Practice and Emergency Medicine, B.P. Koirala Institute of Health Sciences, Dharan, Sunsari, Nepal; 2 Department of Internal Medicine, B.P. Koirala Institute of Health Sciences, Dharan, Sunsari, Nepal

**Keywords:** *emergency departments*, *fever*, *prevalence*

## Abstract

**Introduction:**

Over-the-counter medication use is commonly practised all over the world. However, in a developing country like Nepal, antibiotics form an essential component of OTC drugs. Fever is one of the most common clinical complaints which makes a patient go to the local pharmacy for over- the-counter medication. This study aimed to find out the prevalence of over-the-counter medication use among patients presenting with fever in the Emergency Department in a tertiary care hospital.

**Methods:**

This descriptive cross-sectional study was conducted among patients who visited the Emergency Department with the complaint of at least one episode of documented or undocumented fever after obtaining ethical approval from the Institutional Review Committee. Data collection was conducted from 24 June 2022 to 30 September 2022. Convenience sampling method was used. The point estimate was calculated at a 95% Confidence Interval.

**Results:**

Among 332 patients, 314 (94.58%) (92.14-97.02, 95% Confidence Interval) patients used over- the-counter medication. Antibiotic use was seen in 221 (70.38%) patients.

**Conclusions:**

The prevalence of over-the-counter medication use among patients with fever was found to be higher than the studies conducted in similar studies.

## INTRODUCTION

The use of over-the-counter (OTC) drugs is a common practice worldwide. Around 78.9% of the participants in an online survey had previously taken or were currently taking OTC drugs, half of which consisted of analgesics.^1^ However, the situation is different in developing countries like Nepal where the bulk of OTC drugs consists of antibiotics.^2^ Antimicrobial resistance (AMR) is the most feared complication arising out of this practice. AMR is predicted to cause an estimated loss of 10 million lives and $US100 trillion.^3^ Most of the time patients get such drugs from local pharmacies.^4^

OTC medication use is a huge problem as it can lead to various harmful effects if used without proper supervision. Moreover, the use of antibiotics can lead to antibiotic resistance which can pose a challenge in infection control.

This study aimed to find out the prevalence of over-the-counter medication use among patients presenting with fever in the Emergency Department in a tertiary care hospital.

## METHODS

A descriptive cross-sectional study was conducted in the Emergency Department of B.P. Koirala Institute of Health Sciences (BPKIHS), Dharan, Sunsari, Nepal from 24 June 2022 to 30 September 2022. Ethical approval was obtained from the Institutional Review Committee of the same institute (Reference number: 2251/022). All patients who visited the Emergency Department of BPKIHS with the complaint of at least one episode of documented or undocumented fever within the past seven days were included in the study. Patient below 14 years of age were excluded from the study. Convenience sampling method was used. The sample size was calculated using the following formula:


$$\begin{array}{ll} \text{n} & ={\text{Z}}^{2}\times \frac{\text{p}\times \text{q}}{{\text{e}}^{\text{2}}}\\ & =1.96^{2}\times \frac{0.50\times 0.50}{0.06^{2}}\\ & =267 \end{array}$$

Where,

n = minimum required sample size
Z = 1.96 at 95% Confidence Interval (CI)
p = prevalence taken as 50% for maximum sample size calculation
q = 1-p
e = margin of error, 6%

The calculated sample size was 267. However, 332 samples were taken in our study.

Fever was defined as an axillary temperature ≥38°C.^5^ OTC drug was defined as any drug that was purchased OTC without the prescription of a registered medical practitioner.^6^ The Department of Drug Administration (DDA) of Nepal has also categorised various OTC drugs like paracetamol and ibuprofen into category C and subcategory 2.^7^ They were each given a questionnaire which included demographic data and other information such as duration of fever, associated symptoms, OTC drug use and its duration and source of OTC drugs.

Data were entered and analysis was performed using Microsoft Excel. The point estimate was calculated at a 95% CI.

## RESULTS

Among 332 patients, the prevalence of use of OTC medication was 314 (94.58%) (92.14-97.02, 95% CI). Among 314 patients, antibiotic use was seen in 221 (70.38%) patients. There were 169 (53.82%) males and 145 (46.18%) females. The mean age of the patients was 45.57±18.54 years. A total of 49 (15.61%) patients used only one drug whereas 265 (84.39%) patients used two or more than two drugs. Paracetamol was the second most commonly used OTC drug which was used by 144 (45.85%) patients followed by proton pump inhibitor (PPI) which was used by 136 (43.31%) patients (Table 1).

**Table 1:** OTC use among patients with fever (n = 314).

Drugs	n (%)
Antibiotics	221 (70.38)
Paracetamol	144 (45.86)
Paracetamol-Ibuprofen	123 (39.17)
Proton pump inhibitor	136 (43.31)
Cough syrup	67 (21.34)
Multi-vitamin	58 (18.47)
Paracetamol-Chlorpheniramine-phenylephrine	36 (11.46)
H2 receptor antagonist	24 (7.64)
Other NSAIDs	29 (9.24)
Steroids	20 (6.37)

Among 314, the use of amoxicillin-clavulanate was seen in 82 (26.11%) (Table 2).

**Table 2:** Use of OTC antibiotic drugs (n = 221).

Drugs	n (%)
Amoxicillin-clavulanate	82 (26.11)
Azithromycin	67 (21.34)
Metronidazole	24 (7.64)
Cefixime	55 (17.52)
Ofloxacin	22 (7.01)
Ciprofloxacin	10 (3.18)
Cefpodoxime	16 (5.10)
Miscellaneous	17 (5.41)

## DISCUSSION

In this study, the prevalence of OTC medication use among all fever patients was 314 (94.58%). A study from Thailand reported that 88.2% of the population used OTC drugs.^8^ Another study from Spain using an online questionnaire found that 78.9% out of a total of 728 respondents used OTC drugs.^1^ Yet another study from South India found that 71% of the total 200 respondents had consumed OTC medications on their own.^9^ Similarly a study done in Pokhara, Nepal found that 38.2% of patients used OTC drugs on their own.^10^ Various other studies have shown that 30-60% of Nepal self-medicates.^11^ The main reason for this very high rate of OTC use in our study could be that people have become more apprehensive about the shortage of OTC drugs after the COVID-19 pandemic so they keep reserving OTC drugs for the future. Another reason could be a rapid increase in the number of pharmacies all over the country. Additionally, the widespread availability of the internet leads people to look up minor ailments online and take OTC drugs, which is another important factor.

In our study, use of OTC antibiotic drug was seen in 221 (70.38%). This finding is in sharp contrast to other studies where the prevalence of antibiotic consumption is much lower.^1,8^ It has been seen that antibiotics are taken OTC mostly in low and middle-income countries.^11^ Data show that 30-50% of antibiotics are prescribed unnecessarily in India, China and Kenya.^12^ The reason for such a high rate of antibiotic use in our study could be because of easy access to pharmacies, the widespread assumption among pharmacists and the general public that every case of fever needs antibiotics and the lack of proper policy regarding antibiotic use.

Our study showed that the most commonly used antibiotics were amoxicillin-clavulanate followed by azithromycin. This finding is similar to other studies from Southeast Asia.^13^ This rampant use of antibiotics is a major reason for antimicrobial resistance in this part of the world and could lead to a huge burden of antimicrobial resistance in the future. In this study, paracetamol was found to be the second most commonly used OTC drug. This finding is similar to studies from other countries that have reported nonsteroidal anti-inflammatory drugs (NSAIDs) and paracetamol as the most common OTC drug.^1,5,8^ One study from Nepal also reported that paracetamol is the most commonly used OTC drug.^10^ The reason for this high use of paracetamol could be because it is cheap and easily available in pharmacies. The other reason is that every household has a stack of paracetamol tablets in case of fever or other minor ailments. In our study, PPI was the third most commonly OTC drug which is again similar to the findings from other studies where the use of PPI is second to NSAIDs.^1,8^ This common use of PPI could be because of the widely held belief among people that NSAIDs, paracetamol and antibiotics cause gastritis because of which they self-medicate or get it from pharmacies.

Another finding from our study was the OTC use of steroids which was taken by 6.36%. This could have very harmful consequences both in the short and long term. We were not able to find any study looking at the use of oral steroids as an OTC drug because of which it is difficult to understand the use of steroids in the local or global context. Our study also showed that the use of multiple drugs was more common than the use of a single drug. This could be explained by the fact that a large number of cases had other associated symptoms such as rhinorrhea, cough, and diarrhoea for which they took medications other than paracetamol. The other reason is the excessive use of multivitamins and PPIs sold by local pharmacies for personal profit.

Our study had a few limitations. It was a singlecentered study. The duration of the study was short with a small sample size. Samples were taken based on the patient's history of fever but many patients had not measured their temperatures. Many patients were not literate and could not tell what drugs they were taking prior to presentation which might have resulted in bias.

## CONCLUSIONS

The prevalence of the use of OTC drugs among patients with fever was found to be higher than studies done in similar settings. Better policies on OTC drug sale and their strict implementation from the concerned authorities is much needed.
